# Identifying Cervical Predictors of Recreational Mixed Martial Arts Participation: A Case-Control Study

**DOI:** 10.3390/sports13050155

**Published:** 2025-05-20

**Authors:** Leia Holland, Eleuterio A. Sánchez Romero, Juan Nicolás Cuenca-Zaldívar, Rob Sillevis

**Affiliations:** 1 Department of Rehabilitation Sciences, Florida Gulf Coast University, Fort Myers, FL 33965, USA; lmholland1706@eagle.fgcu.edu; 2 Physiotherapy and Orofacial Pain Working Group, Sociedad Española de Disfunción Craneomandibular y Dolor Orofacial (SEDCYDO), 28009 Madrid, Spain; 3 Research Group in Nursing and Health Care, Puerta de Hierro Health Research Institute-Segovia de Arana (IDIPHISA), 28222 Majadahonda, Spain; nicolas.cuenca@salud.madrid.org; 4 Interdisciplinary Research Group on Musculoskeletal Disorders, 28014 Madrid, Spain; 5 Grupo de Investigación en Fisioterapia y Dolor, Departamento de Fisioterapia, Facultad de Enfermería y Fisioterapia, Universidad de Alcalá, 28801 Alcalá de Henares, Spain; 6 Physical Therapy Unit, Primary Health Care Center “El Abajón”, 28231 Las Rozas de Madrid, Spain

**Keywords:** Mixed Martial Arts, Neck pain, concussion, range of motion, articular, proprioception, muscle strength, physical endurance, recreation, Neck injuries

## Abstract

**Background**: Recreational participation in Mixed Martial Arts (MMA) has rapidly increased. Despite consistent evidence of a high injury prevalence in MMA athletes, the neuromuscular implications of regular MMA training remain underexplored. The cervical spine is particularly vulnerable to trauma due to repetitive impacts and high mechanical demands in combat sports. **Methods**: This case-control study compared cervical spine function and self-reported symptoms between 25 recreational MMA athletes and 25 matched individuals who engaged in general fitness training. Outcome measures included Neck Disability Index (NDI), Post-Concussion Symptom Scale (PCSS), pain and headache reports, cervical range of motion (ROM), proprioception, isometric strength, and endurance. Multivariate logistic regression analysis was used to identify the predictors of group classification. **Results**: The MMA group exhibited significantly higher values for post-concussion symptoms (*p* = 0.012), cervical flexor endurance (*p* = 0.031), and the number of concussions (*p* = 0.001) but lower flexion ROM (*p* = 0.031). No significant differences were observed in strength, proprioception, or NDI scores. Logistic regression identified the number of concussions, age, total cervical ROM, and average rotation strength as significant predictors of group membership (model AUC = 0.96; Nagelkerke R^2^ = 0.797). **Conclusions**: Recreational MMA athletes demonstrated higher rates of concussion-related symptoms and reduced cervical flexion ROM than noncontact exercisers despite no statistically significant differences in strength and proprioception. These findings suggest that cumulative exposure to amateur MMA is associated with alterations in cervical neuromuscular characteristics. These results support the implementation of targeted mobility, endurance, and injury prevention programs in recreational MMA training.

## 1. Introduction

Mixed Martial Arts (MMA) is a full-contact sport that incorporates a variety of combat disciplines, including Brazilian jiu-jitsu, wrestling, and Muay Thai kickboxing [1]. Since gaining widespread popularity in 1993 with the first Ultimate Fighting Championship, MMA has become the fastest-growing sport in the United States [1,2]. Beyond competition, MMA is increasingly being practiced recreationally for fitness, which has been associated with improved cardiovascular and metabolic health outcomes [1]. However, the sport’s physically demanding and collision-prone nature poses a substantial risk of injury. MMA involves grappling, striking, and takedown techniques that often result in musculoskeletal and neuromuscular injuries, particularly to the head and cervical spine [3]. Head and neck injuries account for 38–48% of all reported MMA-related injuries [2].

Previous research has shown that athletes engaged in high-collision sports, including combat sports, often exhibit reduced cervical spine range of motion (ROM), increased cervical muscle strength, and altered proprioception [4,5,6]. Injury risk appears to be inversely related to experience, with less experienced fighters sustaining up to twice as many injuries as their seasoned counterparts [3]. Training environments are associated with more injuries than competitive bouts, with a 4:1 training-to-competition injury ratio [7].

Concussions are particularly prevalent in MMA, where victory is often achieved via knockout [8]. Head trauma contributed to nearly one-third of all UFC fights between 2000 and 2021 [8]. Concussions occur due to rapid acceleration-deceleration of the brain and are influenced by cervical muscle strength and neuromuscular control [9,10,11]. Greater neck strength has been associated with lower concussion risk, with studies showing that each additional pound of neck strength may reduce concussion incidence by 5% [12]. Moreover, imbalances between cervical flexor and extensor strength have been suggested as potential indicators of increased vulnerability [13].

The cervical musculature plays a central role in head stabilization. Strength deficits in deep stabilizing muscles, such as the longus colli and longus capitis, have been linked to cervical dysfunction [14], whereas anticipatory neck muscle activation appears to be protective during sport-related impacts [15]. This has led to calls for cervical strengthening interventions, including strength- and reaction-based training, to mitigate head and neck injury risk in contact sports.

Cervical ROM limitations are also indicative of dysfunction and are commonly observed in whiplash-associated disorders (WAD) [6,16,17]. MMA fighters are exposed to repetitive hyperextension-flexion mechanisms, predisposing them to WAD-like injuries and associated impairments, including pain and decreased mobility [3,17]. These repeated head impacts may also affect cervical proprioception and vestibular function, especially in highly sensorized upper cervical segments [18,19]. These regions are rich in mechanoreceptors with connections to the visual, vestibular, and sympathetic systems and play a key role in head-eye coordination and postural control [19]. Proprioceptive dysfunction in these systems is frequently reported in athletes with neck pain, whiplash, or degenerative cervical changes [19].

Furthermore, cervical rotator muscles are critical in controlling head movement during dynamic tasks and resisting external perturbations in combat situations. Rotational strength has been associated with reduced head acceleration during impact and improved anticipatory activation in collision sports [10,12,14]. In the context of MMA, where grappling, takedowns, and striking maneuvers generate substantial rotational forces, average isometric rotational strength may reflect sport-specific neuromuscular demands and adaptive responses. Therefore, its inclusion as a predictor variable in the regression model is both physiologically and clinically relevant.

Despite this emerging body of evidence, limited data exist on recreational MMA practitioners. While professional fighters have been studied, recreational athletes may differ in exposure intensity, training volume, and physical preparedness. To date, only a few studies have assessed whether these individuals exhibit similar cervical functional adaptations or risk profiles. Understanding these characteristics is critical for informing training and preventive strategies for this rapidly growing population.

Therefore, this study aimed to compare cervical ROM, isometric muscle strength, endurance, proprioception, and self-reported symptoms between recreational MMA athletes and individuals engaged in general fitness training. The secondary objective was to explore potential correlations among clinical and functional variables. These findings may inform future guidelines for injury prevention and performance optimization in recreational martial arts.

## 2. Materials and Methods

### 2.1. Study Design and Participants

This case-control study used convenience sampling to compare differences in neck strength, ROM, and proprioception between recreational MMA athletes and individuals who exercise for general health. Subjects were recruited from 1 May to 30 September 2024, from the students and faculty at Marieb College of Health & Human Services at Florida Gulf Coast University, and members enrolled at The Grounds MMA Academy in Bonita Springs, Florida. The MMA group participants were matched to the general health exercise group by age, sex, weight, height, and hours of training per week. This study was approved by the Florida Gulf Coast University Institutional Review Board (IRB# 2024-30, date: 8 April 2024). In accordance with the Declaration of Helsinki, all participants signed an informed consent form before inclusion and agreed that their study information would be published anonymously.

#### Participants

Based on a power analysis using g*power with an estimated effect size of 0.50, power of 0.80, and 95% confidence interval, 34 subjects were recruited [20]. To be included in this study, subjects were required to be between the ages of 18 and 40 years old. Subjects were excluded from the study if they had a history of cervical spine surgery, active cervical radiculopathy, rheumatologic conditions, known neuromuscular diseases, such as multiple sclerosis, or known vestibular disorders that would impair balance. They were either younger than 18 years or older than 40 years old. Subjects in the control group (CG) were excluded if they had a history of involvement in mixed martial arts and were included if they participated in at least 30 min of activity three times a week. To be included in the MMA group, subjects had at least 2 years of experience and were trained at least 2 times a week. All participants in the experimental group participated in martial arts activities, including Muay Thai kickboxing, Brazilian jiu-jitsu, and/or Krav Maga. The MMA group participants reported training for an average of 9.28 h each week, significantly surpassing the minimum requirement for inclusion.

### 2.2. Outcome Measures

To comprehensively assess the cervical spine function and associated symptoms, the following outcome measures were employed:

#### 2.2.1. Neck Disability

Each participant completed the Neck Disability Index (NDI), a 10-item neck-specific disability questionnaire. Each item is scored between 0 and 5, with a maximum score of 50. Lower scores indicate lower levels of disability. The reliability and validity of the NDI have been demonstrated in evaluating disability due to neck pain [21,22,23,24]. The NDI demonstrates good internal consistency and test-retest reliability without floor or ceiling effects and is proven valid compared to other pain and disability measures [21,23].

#### 2.2.2. Post-Concussion Symptoms

Post-concussion symptoms were assessed using the Post-Concussion Symptom Scale (PCSS), a 22-item symptom checklist used to assess the presence and severity of the symptoms. The PCSS generates two scores: a total score ranging from 0 to 22 and a symptom severity score ranging from 0 to 132. Lower scores indicated lower symptom severity. The PCSS has demonstrated high internal consistency (0.93) and moderate test-retest reliability (0.62–0.69) [25,26,27] and is a reliable method for identifying cognitive deficits following a concussion [28].

#### 2.2.3. Pain and Headaches

Pain intensity was measured using a 10-point Visual Analog Scale (VAS), with higher scores representing greater pain. Headache presence and frequency were documented based on participants’ self-reports, including the number of weekly episodes.

#### 2.2.4. Cervical Proprioception

Cervical proprioception was assessed through the Joint Position Error (JPE) test using a CROM device to measure repositioning accuracy in flexion, extension, side bending, and rotation. This method is widely used in clinical and research settings.

#### 2.2.5. Cervical Range of Motion (CROM)

The CROM was measured in all anatomical planes (flexion, extension, lateral flexion, and rotation) using a CROM device (Performance Attainment Associates, Lindstrom, MN, USA) with triple inclinometers. The participants performed each movement actively, and the maximum angle was recorded.

#### 2.2.6. Cervical Muscle Strength and Endurance

Isometric cervical strength was assessed using a microFET2 Handheld Dynamometer (Hoggan Health Industries, Draper, UT, USA) in six directions: flexion, extension, lateral flexion (left and right), and rotation (left and right). The deep neck flexor endurance test was performed in the supine position following Grimmer’s method, with participants instructed to hold a chin-tuck position above the examiner’s fingers for as long as possible. Familiarization trials and rest intervals were standardized.

### 2.3. Procedures

Next, the subjects underwent a series of assessments to quantify the cervical spine’s ROM, strength, endurance, and proprioception. The total measurement protocol took approximately 20 min. The measures were completed from least to most fatiguing and provided sufficient rest time to allow adequate recovery and minimize the testing effect.

The first assessment measured the cervical range of motion using a CROM measuring device (Performance Attainment Associates™, Lindstrom, MN, USA). The participants actively moved through flexion, extension, side-bending, and rotation. Each participant performed one trial, and the maximum degree was recorded. The CROM device has been shown to be the most reliable and valid tool for measuring cervical ROM [28]. Three inclinometers were used: one in the sagittal plane to measure flexion/extension, one in the frontal plane to measure lateral flexion bilaterally, and another in the horizontal plane to measure cervical rotation [28,29]. The normative values for cervical flexion are around 80°; extension is 50°; cervical lateral flexion is around 45° bilaterally; and cervical rotation is 80° bilaterally [30].

Next, the participants completed the joint position error testing using the CROM measuring device to measure the degree of error. For the joint position error test, the participants were placed in half of the range of motion available for flexion, extension, side bending, and rotation. The participant was asked to remember the position and then recreate it. The degree of error between the first and second positions is recorded. The joint position error test has varying reports of validity, ranging from moderate to weak validity to strong (d = 0.38–0.96) [31].

Isometric strength assessment was performed as follows: Isometric strength was measured using self-generated resistance handheld dynamometry (microFET2™, Hoggan Health Industries, Draper, UT, USA). This measurement protocol was previously reported by Versteegh et al. [31]. High test-retest reliability with an intraclass correlation coefficient of 0.94–0.97 was demonstrated when subjects were seated comfortably without back or armrests for all test positions [32]. The subjects were tested for upper-body strength, cervical flexion, cervical extension, cervical side bending, and cervical rotation. The participant was instructed to “press as hard as they could into the dynamometer without moving their head and hold it for three seconds” during cervical measurements.

Since the assessment of endurance of the cervical flexor muscles is the most fatiguing, this test was performed last. Grimmer originally described the clinical testing of deep cervical flexor endurance, and excellent intratester reliability of 0.92–0.93 has been reported [33,34]. The subjects were placed in the supine position and were shown how to properly perform a chin tuck and raise their head just above the fingertips of the examiner. The subjects were instructed to “*hold this position for as long as possible*”. The test was completed if the subjects decided to end it due to pain or excessive fatigue, lost the chin tuck, or raised or lowered their head excessively. The subjects participated in three practice rounds of less than 10 s to improve test performance. They were given 5 min of rest before the assessment [34].

### 2.4. Statistical Analysis

For statistical analysis, the R program (Ver. 4.1.3) was used (R Foundation for Statistical Computing, Institute for Statistics and Mathematics, Welthandelsplatz 1, 1020 Vienna, Austria). The significance level was set at *p* < 0.05. The Shapiro-Wilk test was used to test the distribution of quantitative variables. Quantitative variables are described as mean ± standard deviation, and qualitative variables as absolute and relative values (%).

The presence of significant differences between the martial arts training group and the general training group in quantitative variables was analyzed using Student’s *t*-test (after checking the assumption of homogeneity of variance using the Levene test) or the Mann-Whitney U test, depending on their distribution, and Fisher’s exact test was used for qualitative variables.

A binary logistic regression model was applied between the dependent variable, type of training (martial arts or general), frequency of concussions, clinical and demographic variables, total range of motion in each anatomical plane, and average strength in lateral flexion and rotation (flexion and extension were analyzed separately because they involved nonsymmetrical muscle groups). The backward stepwise method was used to select the model with the lowest AIC (Akaike Information Criterion (AIC) after eliminating variables with a VIF (Variance Inflation Factor) greater than 5. The coefficients and odds ratios with the corresponding confidence intervals (95% CI) and significance level were calculated from the final model. Goodness of fit was determined by comparing the deviance of the final model with the null deviance, model efficiency, Hosmer–Lemeshow test, and by visual inspection of the model residual graphs. The explained variability was calculated using Nagelkerke’s pseudo R^2^. The significance of each explanatory variable’s contribution to the model was calculated. Receiver operating curve (ROC) analysis was performed to determine the sensitivity, specificity, and Area Under Curve (AUC), and the correct classification rate for each significant variable.

## 3. Results

**Figure 1:**
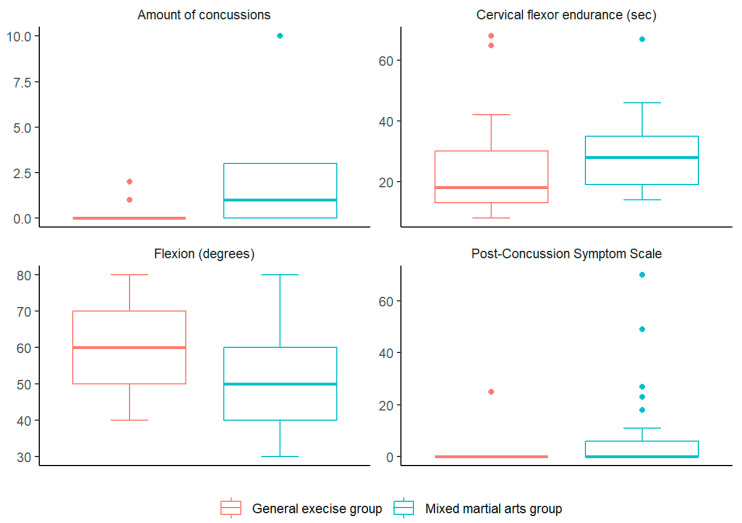
Significant variables box plots.

A total of 50 subjects with an average age of 27.80 ± 5.46 participated in the study, with a predominance of men (72.0%), with no socio-demographic differences between the two groups. Participants in the MMA group reported an average of 9.28 h of training per week, far exceeding the minimum inclusion threshold. On the other hand, the presence of significant differences was verified in the clinical variables Amount of concussions, Post-Concussion Symptom Scale, Cervical flexor endurance (s) amount of concussions (Z = −3.294, *p* < 0.001), Post-Concussion Symptom Scale (Z = 2.521, *p* = 0.012), and cervical flexor endurance (Z = 2.156, *p* = 0.031) with higher values in the Mixed martial arts group [1.68 (0.539, 2.821), 7.2 (−0.262, 14.662), 5.12 (−3.109, 13.349) points of difference compared to the general exercise group, respectively], while in the variable Flexion (°) (Z = −2.156, *p* = 0.031), the values were lower in the Mixed martial arts group [−8 (−15.37, −0.63) points of difference compared to the general exercise group (Table 1, Figure 1).

After eliminating variables with a VIF greater than five and selecting the model with the lowest AIC, the variables number of concussions, Age, Total flexion-extension (°), total rotation (°), total side bending (°), average rotation strength, and Cervical Joint Position Error test were retained as predictors of physical training type. The deviance was 23.81 (42) less than the null of 69.31 (49), and the effectiveness was significant (X2(7) = 45.502, *p* < 0.001), indicating that the model effectively predicted the physical training type. The significant Hosmer-Lemeshow test (X2(8) = 50, *p* < 0.001) indicated an inadequate overall model fit. Nagelkerke pseudo R2 had a high value of 0.797.

The graph with the predicted values shows that the residuals did not exceed the range (−2, 2), indicating a good fit of the final selected model and its adequacy (Supplementary Material, Figure S1a). All values presented a Cook distance of less than 1, compared to the observed values and the leverages, with the absence of influential values outside normal limits. However, there are some atypical values that exist (Supplementary Material Figure S1b,c). The residuals and their standardized versions also did not exceed the band (−2, 2) compared to the adjusted values, indicating a good fit of the model and its adequacy (Supplementary Material Figure S1d,e). The distribution of residuals in the Q-Q plot was not normal (Supplementary Material Figure S1f). Finally, the graph of residuals versus leverage seems to indicate the presence of influential data (Supplementary Material Figure S1b,h), respectively.

The odds ratios indicate that for each point increase in the variables Amount of concussions, Age, and Total rotation (°), the probability of practicing martial arts increased 23.063 (4.253, 524.683), 1.69 (1.23, 2.879), and 1.115 (1.042, 1.246) times, respectively, while for each point increase in the variable Total flexion-extension (°), the probability of practicing martial arts decreased by 0.836 (0.694, 0.927) (Table 2).

**Figure 2:**
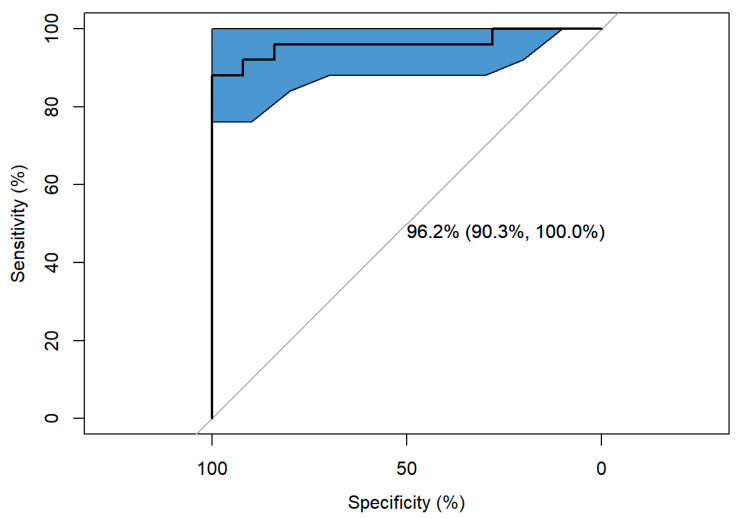
Receiver Operating Characteristic (ROC) curve of the final logistic regression model predicting MMA participation; AUC = 0.96.

The model had a sensitivity of 92% and a specificity of 92%. The ROC curve showed a significant 96.16, 95% CI (90.276%, 100%) (Figure 2).

The significant variables in the model, amount of concussions, age, total flexion-extension (°), and total rotation (°), had a correct classification rate of 70%, 72%, 38%, 62%, and 60%, respectively.

## 4. Discussion

This study used univariate and multivariate analyses to investigate cervical spine function in recreational MMA athletes compared to individuals engaging in general physical activity. Based on the updated statistical model, variables such as the number of concussions, age, total cervical ROM (flexion-extension and rotation), and average rotational strength emerged as significant predictors of group classification, providing new insights into how MMA practice is associated with differences in cervical neuromuscular function.

Although no significant differences were observed in self-perceived disability based on NDI scores, the MMA group reported a significantly higher number of previous concussions and post-concussive symptoms as measured by the PCSS. This aligns with prior reports highlighting the high incidence of head trauma in MMA and its association with subclinical dysfunctions [2,3,7].

Contrary to the prior hypotheses, no statistically significant differences were observed between the groups in isometric cervical muscle strength or deep cervical flexor endurance. Furthermore, the correlation between PCSS and flexor endurance was negligible (r = 0.081), suggesting that strength alone may not serve as a protective factor against concussion in this amateur population. This finding diverges from prior studies involving competitive athletes [9,10,17] and may be explained by the relatively short experience level (average of 2.7 years) and lower training intensity in the current MMA sample [2,3].

Importantly, the logistic regression model demonstrated that a greater cervical rotation range and a higher number of concussions were associated with increased odds of belonging to the MMA group. In contrast, a greater total flexion-extension ROM was negatively associated with MMA practice. This supports earlier findings of limited ROM in high-collision sports and suggests that cervical mobility adaptations may differentiate combat athletes from general exercisers [2,3,4,5,6]. A weak negative correlation between years of practice and cervical ROM (r = −0.257 for flexion; r = −0.208 for extension) reinforces the hypothesis of progressive mobility restrictions related to exposure duration.

Proprioception, measured via joint position error, did not significantly differ between the groups, although a weak inverse relationship was found between rotational mobility and error (r = −0.251). These results may reflect subclinical impairments due to repeated exposure to minor head impacts [19,35,36,37]. Although speculative, this aligns with the concept of cumulative neuromuscular adaptations in the suboccipital system in response to training or trauma.

### Limitations

Several limitations of this study should be considered when interpreting its findings. First, the sample size was modest and recruited through convenience sampling from a single martial arts academy, which may limit the generalizability of the results. Although group matching was applied and a multivariate logistic regression model was used to control for potential confounders, residual confounding cannot be excluded. Moreover, the pronounced group separation observed in Figure 2 may reflect overfitting of the logistic regression model, especially considering the relatively small sample size and the inclusion of multiple predictors. Although diagnostic plots supported model adequacy, the potential for overfitting remains and underscores the need for replication in larger, independent cohorts to confirm model generalizability.

Second, participants in the MMA group practiced various styles (e.g., Muay Thai, Brazilian jiu-jitsu, Krav Maga), each with distinct biomechanical demands. This heterogeneity may have introduced variability in cervical loading patterns and neuromuscular adaptations, potentially masking the style-specific effects. Future studies should stratify participants by martial arts discipline (striking vs. grappling) to better isolate the training-specific impacts.

Another important limitation is the lack of control for potential confounding variables such as previous strength training experience, total exposure to contact or collision sports, and individual recovery practices (e.g., sleep hygiene and equipment use). These factors may influence cervical strength, endurance, and adaptation profiles, and their exclusion may introduce residual bias. Future studies should systematically collect and adjust for these variables to enhance the robustness and ecological validity of predictive models.

Third, most participants had relatively limited martial arts experience (mean = 2.7 years), which may have attenuated the detectable differences in neuromuscular outcomes. It remains unclear whether long-term exposure yields more pronounced deficits or adaptations. Therefore, comparative studies with different levels of experience are warranted.

Fourth, the study did not control for training intensity or frequency beyond a weekly minimum threshold, which could have influenced the outcome measures such as strength and endurance.

Finally, while using the CROM device for measuring joint position error is supported by good reliability [28,31], it differs from the traditional laser pointer method. Although the precision of CROM is acceptable, further research is needed to validate its application, specifically for proprioceptive assessment [31].

## 5. Conclusions

This case-control study compared the cervical functional characteristics of amateur MMA athletes and general exercisers. While muscle strength and proprioception did not differ significantly, the MMA group exhibited higher post-concussion symptomatology, reduced cervical flexion ROM, and a greater number of concussions. Logistic regression revealed that the number of concussions, age, and specific ROM metrics were key predictors of training type, identifying variables strongly associated with MMA participation. This may inform future research toward developing predictive tools for cervical risk monitoring in combat sports, pending external validation.

Although amateur MMA athletes showed indications of strength adaptations without statistically significant group differences, they did not appear to confer protective effects against symptoms. The observed reduction in ROM and increase in concussions emphasize the need for targeted preventive strategies. Based on the observed associations, it may be beneficial to explore the inclusion of cervical mobility, strength, and proprioception training in recreational MMA programs. However, further longitudinal studies are needed to confirm their potential protective role.

Future research should validate these findings in larger cohorts, consider separating groups by martial arts style (striking vs. grappling), and employ longitudinal designs to assess cervical function progression over time.

## Figures and Tables

**Table 1:** Clinical and demographic characteristics of the participants.

		Overall	Mixed Martial Arts Group	General Execise Group	Levene’s Test (^a^ *p* Value)	Average Difference between Groups (95%CI)	^a^ *p* Value
n		50	25	25	NA		NA
Socio-demographic characteristics							
Age		27.80 ± 5.46	29.20 ± 6.34	26.40 ± 4.06	NA	−3 (−6, 1)	0.149
Gender, n (%)	Female	14 (28.0)	5 (20.0)	9 (36.0)	NA	—	0.345
	Male	36 (72.0)	20 (80.0)	16 (64.0)	NA	—	NA
Weight (kg)		78.58 ± 12.22	78.75 ± 10.06	78.41 ± 14.26	0.156	0 (−6.795, 6.795)	0.922
Height (mtrs)		1.76 ± 0.08	1.76 ± 0.08	1.76 ± 0.08	NA	0 (−0.051, 0.051)	0.868
Body Mass Index		25.23 ± 3.18	25.35 ± 2.62	25.11 ± 3.70	0.059	−0.184 (−1.924, 1.549)	0.794
Hours training per week		7.98 ± 5.40	9.28 ± 6.22	6.68 ± 4.17	NA	−2 (−5, 0)	0.119
Years of martial arts training		—	5.30 ± 4.78	—	NA	−4 (−5, −3)	NA
Clinical characteristics							
Amount of concussions		1.04 ± 2.12	1.88 ± 2.73	0.20 ± 0.50	NA	−1 (−2, −0.001)	0.001
Post-Concussion Symptom Scale		4.60 ± 13.25	8.20 ± 17.51	1.00 ± 5.00	NA	−6 (−8, −0.001)	0.012
Neck pain, n (%)	No	13 (26.0)	9 (36.0)	4 (16.0)	NA	—	0.196
	Yes	37 (74.0)	16 (64.0)	21 (84.0)	NA	—	NA
10 points Visual Analog Scale		5.94 ± 12.52	8.48 ± 14.90	3.40 ± 9.21	NA	0 (0, 0)	0.11
Headaches, n (%)	No	12 (24.0)	7 (28.0)	5 (20.0)	NA	—	0.742
	Yes	38 (76.0)	18 (72.0)	20 (80.0)	NA	—	NA
Number of headaches per week		0.48 ± 0.97	0.48 ± 0.87	0.48 ± 1.08	NA	0 (0, 0)	0.631
Neck Disability Index		2.52 ± 3.06	2.84 ± 2.91	2.20 ± 3.23	NA	0 (−2, 0)	0.31
Cervical flexor endurance (sec)		26.28 ± 14.53	28.84 ± 12.40	23.72 ± 16.23	NA	−6 (−14, −1)	0.031
Calibration (kg)		34.84 ± 18.08	36.39 ± 18.29	33.29 ± 18.11	NA	−2.8 (−12.8, 6)	0.461
Neck muscles strength							
Flexion (kg)		15.99 ± 7.05	17.45 ± 7.64	14.52 ± 6.21	0.271	−2.8 (−7.1, 1.1)	0.144
Extension (kg)		20.49 ± 6.71	21.83 ± 7.08	19.14 ± 6.17	0.21	−2.8 (−6.5, 1.3)	0.158
Right rotation (kg)		10.25 ± 3.96	10.79 ± 3.40	9.71 ± 4.46	0.227	−1.5 (−3.5, 1.1)	0.34
Left rotation (kg)		10.13 ± 4.18	10.37 ± 3.77	9.90 ± 4.62	NA	−0.5 (−3.2, 1.8)	0.655
Right side bending (kg)		11.20 ± 4.37	10.91 ± 4.13	11.49 ± 4.66	0.473	0.5 (−2.1, 3.3)	0.646
Left side bending (kg)		11.55 ± 4.14	11.56 ± 3.85	11.54 ± 4.50	0.482	−0.1 (−2.6, 2.6)	0.987
Neck range of movement							
Flexion (°)		56.40 ± 13.44	52.40 ± 13.93	60.40 ± 11.90	NA	10 (<0.001, 20)	0.031
Extension (°)		66.60 ± 12.31	66.60 ± 13.29	66.60 ± 11.52	NA	0 (−10, 10)	0.75
Right rotation (°)		64.30 ± 14.25	66.60 ± 12.81	62.00 ± 15.48	NA	−5 (−15, 0)	0.23
Left rotation (°)		64.50 ± 13.64	67.20 ± 12.08	61.80 ± 14.78	NA	−5 (−15, 0)	0.145
Right side bending (°)		44.40 ± 9.40	45.60 ± 9.61	43.20 ± 9.23	NA	0 (−10, 0)	0.418
Left side bending (°)		47.50 ± 8.88	48.80 ± 9.27	46.20 ± 8.45	NA	0 (−10, 0)	0.366

Data are expressed as mean ± standard deviation or as absolute and relative absolute values (%); 95%CI: 95% confidence interval. ^a^ significant if *p* < 0.05 (shown in red).

**Table 2:** Final model summary.

	Odds Ratio (95%CI)	Coefficient (SE)	95%CI	Z Value	^a^ *p* Value	Variable Importance
(Intercept)	0 (0, 4.695)	−10.29 (6.856)	−26.42, 1.547	−1.501	0.133	
Amount of concussions	23.063 (4.253, 524.683)	3.138 (1.141)	1.448, 6.263	2.750	0.006	2.75
Age	1.69 (1.23, 2.879)	0.525 (0.207)	0.207, 1.058	2.530	0.011	2.53
Total flexion-extension (°)	0.836 (0.694, 0.927)	−0.179 (0.07)	−0.365, −0.076	−2.575	0.01	2.575
Total rotation (°)	1.115 (1.042, 1.246)	0.109 (0.043)	0.041, 0.22	2.524	0.012	2.524
Total side bending (°)	1.085 (1.004, 1.212)	0.081 (0.046)	0.004, 0.192	1.771	0.077	1.771
Average rotation strength (kg)	0.686 (0.41, 0.964)	−0.378 (0.207)	−0.891, −0.037	−1.823	0.068	1.823
Cervical Joint Position Error test	0.853 (0.649, 1.016)	−0.159 (0.108)	−0.432, 0.016	−1.482	0.138	1.482

95%CI: 95% confidence interval; SE: Standard Error. ^a^ significant if *p* < 0.05 (shown in red).

## Data Availability

The original contributions presented in the study are included in the article, and further inquiries can be directed to the corresponding author.

## Supplementary Materials

The following supplementary material is available at: https://www.mdpi.com/article/10.3390/sports13050155/s1: Figure S1. Diagnostic Plots for the Logistic Regression Model: (a–h) Includes residuals analysis, Cook’s distance, leverage, standardized residuals, Q-Q plot, and ROC curve for the final binary logistic regression model predicting group classification (MMA vs. control). These plots support the adequacy and assumptions of the statistical model applied in the study.

## Abbreviations

The following abbreviations are used in this manuscript:

MMA	Mixed Martial Arts
ROM	Range of Motion
WAD	Whiplash Associated Disorder
CG	Control group
MAG	Martial Arts group
NDI	Neck Disability Index
PCSS	Post Concussion Symptom Scale
