# Supplementary figures and images for: Identifying Cervical Predictors of Recreational Mixed Martial Arts Participation: A Case-Control Study
Source: Sports (Basel). 2025 May 20;13(5):155. doi: 10.3390/sports13050155 (PMC12115610; doi:10.3390/sports13050155)

## Supplementary Material

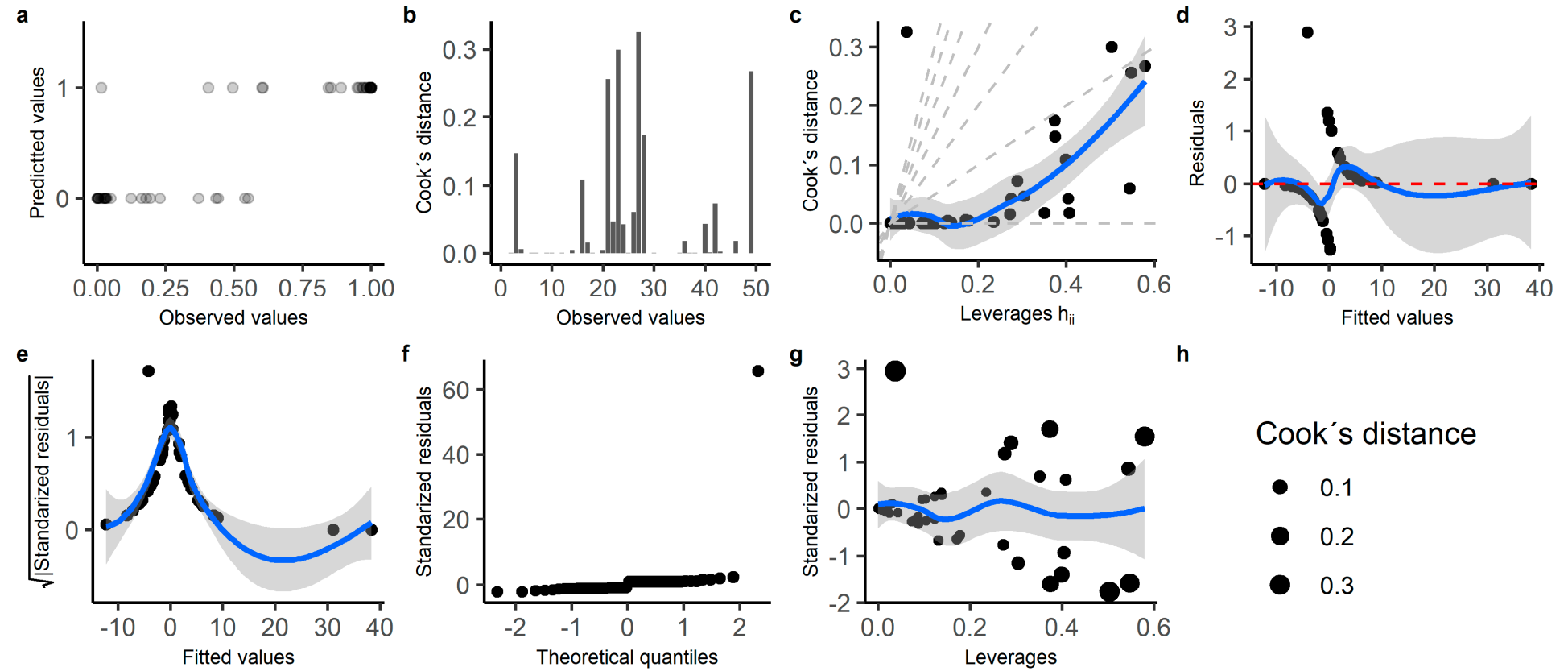

**Figure S1.** Plot diagnosis for training type models.

Supplement: Supplementary file 1 [file sports-13-00155-s001.zip › sports-3635925-supplementary.pdf]
